# The impact of gender and parenthood on physicians' careers - professional and personal situation seven years after graduation

**DOI:** 10.1186/1472-6963-10-40

**Published:** 2010-02-18

**Authors:** Barbara Buddeberg-Fischer, Martina Stamm, Claus Buddeberg, Georg Bauer, Oliver Hämmig, Michaela Knecht, Richard Klaghofer

**Affiliations:** 1 Department of Psychosocial Medicine, Zurich University Hospital, Zurich, Switzerland; 2 Division of Public and Organizational Health, Institute of Social and Preventive Medicine, Zurich University, Zurich, Switzerland

## Abstract

**Background:**

The profile of the medical profession is changing in regard to feminization, attitudes towards the profession, and the lifestyle aspirations of young physicians. The issues addressed in this study are the careers of female and male physicians seven years after graduation and the impact of parenthood on career development.

**Methods:**

Data reported originates from the fifth assessment (T5) of the prospective SwissMedCareer Study, beginning in 2001 (T_1_). At T5 in 2009, 579 residents (81.4% of the initial sample at T1) participated in the questionnaire survey. They were asked about occupational factors, career-related factors including specialty choice and workplace, work-life balance and life satisfaction. The impact of gender and parenthood on the continuous variables was investigated by means of multivariate and univariate analyses of variance; categorical variables were analyzed using Chi-square tests.

**Results:**

Female physicians, especially those with children, have lower rates of employment and show lower values in terms of career success and career support experiences than male physicians. In addition, parenthood has a negative impact on these career factors. In terms of work-life balance aspired to, female doctors are less career-oriented and are more inclined to consider part-time work or to continue their professional career following a break to bring up a family. Parenthood means less career-orientation and more part-time orientation. As regards life satisfaction, females show higher levels of satisfaction overall, especially where friends, leisure activities, and income are concerned. Compared to their male colleagues, female physicians are less advanced in their specialty qualification, are less prone to choosing prestigious surgical fields, have a mentor less often, more often work at small hospitals or in private practice, aspire less often to senior hospital or academic positions and consider part-time work more often. Any negative impact on career path and advancement is exacerbated by parenthood, especially as far as women are concerned.

**Conclusion:**

The results of the present study reflect socially-rooted gender role stereotypes. Taking into account the feminization of medicine, special attention needs to be paid to female physicians, especially those with children. At an early stage of their career, they should be advised to be more proactive in seeking mentoring and career-planning opportunities. If gender equity in terms of career chances is to be achieved, special career-support measures will have to be provided, such as mentoring programs, role models, flexitime and flexible career structures.

## Background

The profile of the medical profession is changing - not only with the increase in the proportion of female doctors, but also with changes in employment conditions, attitudes towards the profession and the life style of young doctors [1,2]. The percentage of females graduating from medical school has increased by up to 60 percent in most Western countries [3]. Looking back over the decades of the last century, it is noticeable that female doctors were often not married or, if they were married, they tended to have fewer children than their male counterparts. If they did have a family, they often did not specialize, cutting back on their professional career or opting for a career break potentially of several years' duration [4,5]. Nowadays, most female doctors complete a specialty qualification, live in a relationship with a partner and have children as often as their male colleagues; they do not drop out after childbirth, but continue working, although in most cases part-time [6,7]. Gender equality in terms of equal numbers of men and women entering and graduating from medical school has been more than achieved. However, gender equity as regards fairness and justice in structuring professional opportunities, i.e. further career steps and prestigious positions, is far from being realized [8,9]: female physicians are still underrepresented in a number of prestigious disciplines and in the higher echelons of academic and hospital medicine [5]. A number of possible obstacles to career goals that may be presumed to act synergistically include domestic responsibilities, absence of flexibility in career structures and lack of role models [3,10].

Besides feminization, there is a further phenomenon that exerts a major influence on medicine as a profession: the priority given to work and career is decreasing as far as the younger generation of physicians is concerned [11]. Not only women, but also men are increasingly matching their choice of specialty and their career preferences to a controllable life style that allows a good work-family balance [12]. Furthermore, it may be assumed that parenthood has a different gender-related influence on careers, work-life balance and life satisfaction.

From the literary references in the bibliography, it would appear that feminization of medicine and parenthood are two key factors influencing careers in medicine. As a result, one objective of the prospective SwissMedCareer Survey [13] has been to acquire detailed knowledge of characteristics in the career paths of the younger generation of physicians, taking into account the changing profile of the medical profession. *The following issues are addressed: *(1) What is the impact of gender and parenthood on career development as regards employment, career-related factors, work-life balance aspired to and life satisfaction; (2) does parenthood have a different influence on these variables depending on gender; and (3) what sort of careers are female and male physicians with and without children engaged in seven years after graduation regarding specialty choice, workplace and career-aspired to?

## Methods

### Study design and study sample

The study is an ongoing prospective survey of a cohort of graduates of the three medical schools in German-speaking Switzerland (Basel, Berne, and Zurich)*(SwissMedCareer Study)*, beginning in 2001 (T1) [13]. Subjects were re-evaluated every two years. The present paper refers to the results of the fifth assessment (T5), conducted in 2009, seven years after graduation.

To ensure participants' anonymity, the returned questionnaires were only identified by a code. The respondents sent their addresses to an independent address-administration office, allowing for follow-up. The study was approved by the ethical committee of Zurich University.

The *study sample *consists of 579 physicians (292 females, 50.4%; 287 males, 49.6%) participating at T5 with a full data set (81.4% of the initial sample at T1), excluded were 6 physicians not working in a medical field. Twenty-two female and five male physicians are actually taking a break from work, mainly because of children. The mean age of the participants is 35.1 years (SD 2.2 years, range 31 - 50 years). Of the respondents 508 (88.0%) have a stable partnership, of whom 285 are married. Of 180 (35.3%) participants the partner is also a physician. One hundred and one (34.6%) of the females, and 110 (38.5%) of the males have children. There is a tendency of male doctors having more often two and more children compared to female doctors (p = 0.051).

### Instruments

All instruments are self-assessment scales. In the following, it is described what constructs are measured by the instruments:

*- Questions concerning socio-demographic data*,

- Employment in percent of fulltime (100%)

- Choice of medical specialty (Primary Care, Internal Medicine, Surgical fields, Gynecology & Obstetrics, Anesthesiology, Pediatrics, Psychiatry, other specialties such as Ophthalmology, Oto-Rhino-Laryngology, Pathology, Radiology)

- Workplace (Regional hospital, Cantonal hospital, University Hospital, Research Institute, family medicine practice, specialist practice, others such as Health Administration, Medical Informatics, Public Health Institutions)

- Career aspired to (family medicine practice, specialist practice, hospital career without a senior position (Spitalarzt), hospital career with senior positions or academic career)

*- Career Success Scale *[14] is a measure of objective career steps consisting of 7 items addressing scientific activities (research projects, lectures, publications, grants, criteria which correspond to the requirements for tenure track).

*- Subjective Assessment of Career Success *[15] is a measure of one's own career advancement compared to that of other cohort subjects (7-point Likert scale, 1 = less successful - 7 = more successful).

*- Question on Satisfaction with Career Success *[13] (7-point Likert scale, 1 = very unsatisfied - 7 = very satisfied) is a measure of a person's satisfaction with his/her career success.

*- Mentor-Protégé Relationships Questionnaire *[16] (Likert scale 0 - 4) consists of five scales measuring different types of career-support. We used the *Networking Scale *(4 items) and the *Support in career planning *scale (3 items). These two scales describe crucial aspects of mentoring. Our data analyses show that the two scales are highly correlated (r = 0.71). We therefore combined them into one scale named '*Mentoring-Experience Scale*', having a high Cronbach's alpha = 0.93. We further used the *Emotional Support Scale *(4 items).

*- Question on Satisfaction with Career Support *[13] (7-point Likert scale, 1 = very unsatisfied - 7 = very satisfied).

*- Work-life Balance *(5-point Likert scale) [17] investigates which of the four different models of work-life balance (career orientation, balance of work and personal life, part-time orientation, three-phase orientation: work-family break-work) the participants want to experience within 5 years.

*- Life-Satisfaction Questionnaire (Fragebogen zur Lebenszufriedenheit FLZ*^*M*^) (5-point Likert scale) [18,19] is a measure that assesses the satisfaction with 8 life areas (friends/acquaintances, leisure/hobbies, health, income/financial security, occupation/work, housing/living condition, family life/children and partner relationship/sexuality).

### Statistical analyses

All analyses were carried out with SPSS for windows release 15. Descriptive statistics are given in terms of means and standard deviations, counts and percentages respectively. The impact of gender and parenthood on the continuous variables employment, career success and career support, work-life balance and life satisfaction, was investigated by separate two-factorial multivariate and univariate analyses of variance; F- and p-values are reported as well as partial eta-squared for estimation of effect size. Categorical variables such as career steps, specialty choice, workplace, and career aspired to were analyzed with Chi-square tests.

## Results

Based on the main issues of the present study, data analyses focus on *gender specific careers and the impact of parenthood on the careers of female and male physicians*.

Only statistically significant results are described below.

### Employment (Table 1)

The rate of employment for female physicians is lower than for males; physicians with children have a lower rate of employment than those without children.

**Table 1:** Means (SD) of fulltime employment in female and male physicians.

	Female physicians			Male physicians					
EMPLOYMENT	Without children (n = 191) Mean (SD)	With children (n = 101) Mean (SD)	Total (n = 292) Mean (SD)	Without children (n = 177) Mean (SD)	With children (n = 110) Mean (SD)	Total (n = 287) Mean (SD)			
Employment in % of fulltime (100%)	96.0 (12.4)	64.2 (24.6)	85.1 (23.1)	98.6 (6.9)	95.2 (13.2)	97.3 (10.0)			

**Analysis of variance**	**Gender**			**Children**			**Gender × Children**		
	F(1,570)	p	partial eta^2^	F(1,570)	p	partial eta^2^	F(1,570)	p	partial eta^2^

	183.52	<0.001	0.24	201.15	<0.001	0.26	131.12	<0.001	0.19

Results of univariate analysis (N = 579)

### Career success and support (Table 2)

In multivariate analysis, the values established for females in terms of career success and support are lower than for males; this may to be ascribed to lower values in objective and subjectively assessed career success and mentoring experience as found in univariate analyses; overall (multivariate) parenthood generates lower ratings in relation to these career factors apart from objective career success (univariate); the significant interaction between gender and parenthood reveals that females with children have the lowest values in the subjectively assessed career-success ratings. Furthermore, the univariate analysis shows that there is no significant difference between males and females when it comes to satisfaction with their own career; physicians with children demonstrate significantly lower values in terms of career satisfaction compared to those without children.

**Table 2:** Means (SD) of career success and career support in female and male physicians.

	Female physicians			Male physicians					
CAREER SUCCESS AND SUPPORT	Without children (n = 191) Mean (SD)	With children (n = 101) Mean (SD)	Total (n = 292) Mean (SD)	Without children (n = 177) Mean (SD)	With children (n = 110) Mean (SD)	Total (n = 287) Mean (SD)			
Objective career success	1.63 (2.00)	1.12 (1.72)	1.46 (1.91)	2.74 (2.89)	2.93 (3.20)	2.81 (3.01)			
Subjective career success	4.53 (1.20)	3.51 (1.54)	4.18 (1.41)	4.61 (1.14)	4.53 (1.20)	4.58 (1.16)			
Satisfaction with own career	5.44 (1.13)	5.05 (1.40)	5.31 (1.24)	5.38 (1.12)	5.27 (1.46)	5.34 (1.26)			
Mentoring experience	1.76 (1.07)	1.39 (0.94)	1.64 (1.04)	2.03 (1.01)	1.89 (1.05)	1.98 (1.03)			
Satisfaction with career support	4.23 (1.63)	3.75 (1.44)	4.07 (1.58)	4.32 (1.58)	4.18 (1.61)	4.26 (1.59)			

**Analyses of variance**	**Gender**			**Children**			**Gender × Children**		
**multivariate**	F(5,555)	p	partial eta^2^	F(5,555)	p	partial eta^2^	F(5,555)	p	partial eta^2^

	12.46	<0.001	0.10	5.47	<0.001	0.05	4.03	0.001	0.04

**univariate**	F(1,559)	p	partial eta^2^	F(1,559)	p	partial eta^2^	F(1,559)	p	partial eta^2^

Objective career success	43.91	<0.001	0.07	0.50	0.478	<0.01	2.48	0.116	<0.01
Subjective career success	25.40	<0.001	0.04	22.29	<0.001	0.04	18.61	<0.001	0.03
Satisfaction with own career	0.25	0.471	<0.01	5.31	0.022	0.01	1.60	0.207	<0.01
Mentoring experience	18.00	<0.001	0.03	7.80	0.005	0.01	1.59	0.207	<0.01
Satisfaction with career support	3.42	0.065	0.01	4.98	0.026	0.01	1.46	0.228	<0.01

Results of multivariate and univariate analyses (N = 579)

### Work-life balance (Table 3)

A multivariate analysis reveals major differences between females and males when it comes to assessments: females are less career-oriented, aspire more to a balance between work and personal life, are more inclined to consider part-time work or to continue their professional career following a break to start a family (break in their professional career because of family obligations, three-phase orientation) (univariate). Similar differences are evident in relation to the 'parenthood' factor: physicians with children are less career-oriented and more inclined to consider part-time work or to continue their professional career after a break to bring up a family. The three-phase orientation option tends to be considered mostly by females with children, whilst the lowest level of consideration is accorded to career-orientation.

**Table 3:** Means (SD) of work-life balance in female and male physicians.

	Female physicians			Male physicians					
WORK-LIFE BALANCE	Without children (n = 191) Mean (SD)	With children (n = 101) Mean (SD)	Total (n = 292) Mean (SD)	Without children (n = 177) Mean (SD)	With children (n = 110) Mean (SD)	Total (n = 287) Mean (SD)			
Career orientation	2.47 (1.19)	1.65 (0.94)	2.18 (1.17)	3.19 (1.26)	3.06 (1.37)	3.14 (1.30)			
Balance of work and personal life	4.68 (0.55)	4.70 (0.67)	4.69 (0.60)	4.51 (0.79)	4.62 (0.62)	4.55 (0.73)			
Part-time orientation	4.35 (1.01)	4.83 (0.51)	4.52 (0.90)	3.46 (1.39)	3.73 (1.34)	3.56 (1.38)			
Three-phase orientation	3.16 (1.31)	3.97 (1.19)	3.44 (1.33)	2.36 (1.08)	2.56 (1.14)	2.44 (1.10)			

**Analyses of variance**	**Gender**			**Children**			**Gender × Children**		
**multivariate**	F(4,568)	p	partial eta^2^	F(4,568)	p	partial eta^2^	F(4,568)	p	partial eta^2^

	43.06	<0.001	0.23	8.12	<0.001	0.05	4.41	0.002	0.03

**univariate**	F(1,570)	p	partial eta^2^	F(1,570)	p	partial eta^2^	F(1,570)	p	partial eta^2^

Career orientation	103.32	<0.001	0.15	19.00	<0.001	0.03	10.91	0.001	0.02
Balance of work and personal life	4.79	0.029	<0.01	1.25	0.265	<0.01	0.66	0.416	<0.01
Part-time orientation	100.13	<0.001	0.15	14.12	<0.001	0.02	1.02	0.312	<0.01
Three-phase orientation	114.30	<0.001	0.17	23.61	<0.001	0.04	8.91	0.003	0.02

Results of multivariate and univariate analyses (N = 579)

### Life-satisfaction (Table 4)

Overall (multivariate) female physicians show higher life satisfaction than their male colleagues, especially in the domains 'friends', 'leisure activities' and 'income' (univariate); parenting physicians assess their life satisfaction differently (multivariate) due to higher rating of 'family life' (univariate). The multivariate analysis reveals no significant interaction between gender and parenthood.

**Table 4:** Means (SD) of life satisfaction in female and male physicians.

	Female physicians			Male physicians					
LIFE SATISFACTION	Without children (n = 191) Mean (SD)	With children (n = 101) Mean (SD)	Total (n = 292) Mean (SD)	Without children (n = 177) Mean (SD)	With children (n = 110) Mean (SD)	Total (n = 287) Mean (SD)			
Friends	3.58 (1.04)	3.82 (1.09)	3.66 (1.06)	3.48 (1.07)	3.34 (1.12)	3.43 (1.08)			
Leisure activities	3.05 (1.05)	3.12 (1.03)	3.07 (1.04)	3.05 (1.03)	2.64 (1.08)	2.89 (1.07)			
Health	3.75 (1.05)	3.83 (1.11)	3.78 (1.07)	3.84 (1.01)	3.66 (1.12)	3.77 (1.06)			
Income	3.50 (1.07)	3.45 (1.10)	3.48 (1.08)	3.17 (1.16)	3.15 (1.20)	3.16 (1.77)			
Work	3.49 (0.98)	3.57 (1.00)	3.52 (0.97)	3.54 (0.95)	3.75 (0.97)	3.62 (0.96)			
Living condition	4.12 (0.88)	4.10 (0.97)	4.11 (0.91)	4.03 (0.97)	4.04 (0.85)	4.03 (0.92)			
Family life	3.38 (1.26)	4.46 (0.81)	3.76 (1.23)	3.45 (1.12)	4.32 (0.96)	3.79 (1.14)			
Partnership/Sexuality	3.64 (1.27)	3.73 (1.17)	3.67 (1.23)	3.55 (1.34)	3.65 (1.07)	3.59 (1.24)			

**Analyses of variance**	**Gender**			**Children**			**Gender × Children**		
**multivariate**	F(8,559)	p	partial eta^2^	F(8,559)	p	partial eta^2^	F(8,559)	p	partial eta^2^

	3.73	<0.001	0.05	21.71	<0.001	0.24	1.50	0.153	0.02

**univariate**	F(1,566)	p	partial eta^2^	F(1,566)	p	partial eta^2^	F(1,566)	p	partial eta^2^

Friends	9.23	0.002	0.02	0.18	0.675	<0.01	4.09	0.044	0.01
Leisure activities	6.23	0.013	0.01	3.86	0.050	0.01	6.43	0.011	0.01
Health	0.14	0.711	<0.01	0.41	0.523	<0.01	1.83	0.177	<0.01
Income	9.87	0.002	0.02	0.18	0.673	<0.01	0.03	0.872	<0.01
Work	1.88	0.171	<0.01	2.98	0.085	<0.01	0.64	0.426	<0.01
Living condition	0.90	0.342	<0.01	0.01	0.952	<0.01	0.03	0.873	<0.01
Family life	0.12	0.734	<0.01	104.94	<0.001	0.16	1.26	0.262	<0.01
Partnership/Sexuality	0.61	0.434	<0.01	0.76	0.384	<0.01	0.01	0.958	<0.01

Results of multivariate and univariate analyses (N = 579)

### Career steps/Mentoring (Table 5)

Female physicians have a mentor significantly less frequently. There are fewer instances of physicians who have children completing their dissertation. As regards specialty qualification, female physicians with children are less advanced.

**Table 5:** Differences in career- and workplace-related factors between female and male physicians with and without children

	Females (n = 292)	Males (n = 287)		Physicians without children (n = 368)	Physicians with children (n = 211)		Total (n = 579)	Female physicians (n = 292)			Male physicians (n = 287)		
								Without children (n = 191)	With children (n = 101)		Without children (n=177)	With children (n = 110)	
	n (%)	n (%)	p	n (%)	n (%)	p	n (%)	n (%)	n (%)	p	n (%)	n (%)	p
**CAREER STEPS/MENTOR**													
Dissertation (yes)	245 (83.9)	229 (80.4)	0.278	**312 (84.8)**	**162 (77.5)**	**0.032**	474 (82.1)	166 (86.9)	79 (78.2)	0.066	146 (82.5)	83 (76.9)	0.283

Specialty qualification	121 (41.4)	114 (40.1)	0.850	158 (43.1)	77 (36.8)	0.102	235 (40.8)	**90 (47.1)**	**31 (30.7)**	**0.025**	68 (38.6)	46 (42.6)	0.128

Mentor (yes)	**118 (40.5)**	**169 (58.9)**	**<0.001**	188 (51.2)	99 (46.9)	0.342	287 (49.7)	84 (44.2)	34 (33.7)	0.103	104 (58.8)	65 (59.1)	0.999

**SPECIALTY CHOICE**			**<0.001**			**0.020**				0.116			0.137
Primary Care	34 (11.6)	31 (10.8)		**33 (9.0)**	**32 (15.2)**		65 (11.2)	18 (9.4)	16 (15.8)		15 (8.5)	16 (14.5)	
Internal Medicine	93 (31.8)	81 (28.2)		114 (31.0)	60 (28.4)		174 (30.1)	60 (31.4)	33 (32.7)		54 (30.5)	27 (24.5)	
Surgical fields	**15 (5.1)**	**56 (19.5)**		41 (11.1)	30 (14.2)		71 (12.3)	11 (5.8)	4 (4.0)		30 (16.9)	26 (23.6)	
Gynecology & Obstetrics	**28 (9.6)**	**4 (1.4)**		23 (6.3)	9 (4.3)		32 (5.5)	21 (11.0)	7 (6.9)		2 (1.1)	2 (1.8)	
Anesthesiology	23 (7.9)	26 (9.1)		**37 (10.1)**	**12 (5.7)**		49 (8.5)	18 (9.4)	5 (5.0)		19 (10.7)	7 (6.4)	
Pediatrics	**34 (11.6)**	**8 (2.8)**		25 (6.8)	17 (8.1)		42 (7.3)	22 (11.5)	12 (11.9)		3 (1.7)	5 (4.5)	
Psychiatry	24 (8.2)	18 (6.3)		26 (7.1)	16 (7.6)		42 (7.3)	13 (6.8)	11 (10.9)		13 (7.3)	5 (4.5)	
Other specialties	**24 (8.2)**	**48 (16.7)**		**54 (14.7)**	**18 (8.5)**		72 (12.4)	20 (10.5)	4 (4.0)		34 (19.2)	14 (12.7)	
Not decided	17 (5.8)	15 (5.2)		**15 (4.1)**	**17 (8.1)**		32 (5.5)	8 (4.2)	9 (8.9)		7 (4.0)	8 (7.3)	

**WORKPLACE**			**0.005**			**0.006**				**<0.001**			0.356
Regional hospital	42 (14.4)	30 (10.5)		44 (12.0)	28 (13.3)		72 (12.5)	30 (15.8)	12 (11.9)		14 (7.9)	16 (14.5)	
Cantonal hospital	80 (27.5)	92 (32.1)		**121 (33.0)**	**51 (24.2)**		172 (29.8)	**65 (34.2)**	**15 (14.9)**		56 (31.6)	36 (32.7)	
University Hospital	**92 (31.6)**	**109 (38.0)**		**133 (36.2)**	**68 (32.2)**		201 (34.8)	62 (32.6)	30 (29.7)		71 (40.1)	38 (34.5)	
Research Institution	**2 (0.7)**	**10 (3.5)**		10 (2.7)	2 (0.9)		12 (2.1)	2 (1.1)	0		8 (4.5)	2 (1.8)	
Family Medicine practice	**17 (5.8)**	**8 (2.8)**		15 (4.1)	10 (4.7)		25 (4.3)	**9 (4.7)**	**8 (7.9)**		6 (3.4)	2 (1.8)	
Medical specialty practice	8 (2.7)	5 (1.7)		7 (1.9)	6 (2.8)		13 (2.2)	4 (2.1)	4 (4.0)		3 (1.7)	2 (1.8)	
others	**50 (17.1)**	**33 (11.5)**		**37 (10.0)**	**46 (21.8)**		83 (14.4)	**19 (9.5)**	**32 (31.7)**		19 (10.8)	33 (11.5)	

**CAREER ASPIRED TO**			**<0.001**			**0.005**				**0.002**			0.280
Family Medicine practice	50 (17.1)	44 (15.4)		**45 (12.3)**	**49 (23.2)**		94 (16.3)	**24 (12.6)**	**26 (25.7)**		22 (12.6)	23 (20.9)	
Medical Specialty practice	65 (22.3)	57 (20.0)		74 (20.2)	48 (22.7)		122 (21.1)	**36 (18.8)**	**29 (28.7)**		37 (21.1)	19 (17.3)	
Hospital doctor (Spitalarzt)	**32 (11.0)**	**10 (3.5)**		30 (8.2)	12 (5.7)		42 (7.3)	22 (11.5)	10 (9.9)		8 (4.6)	2 (1.8)	
Hospital career (senior/chief physician)	**86 (29.5)**	**109 (38.2)**		**140 (38.3)**	**55 (26.1)**		195 (33.8)	**71 (37.2)**	**15 (14.9)**		69 (39.4)	40 (36.4)	
Academic career	**8 (2.7)**	**37 (13.0)**		29 (7.9)	16 (7.6)		45 (7.8)	6 (3.1)	2 (2.0)		23 (13.1)	14 (12.7)	
Others	15 (5.1)	12 (4.3)		14 (3.8)	13 (6.1)		27 (4.6)	9 (4.7)	6 (5.3)		5 (2.9)	12 (4.3)	
Not decided	**36 (12.3)**	**16 (5.6)**		34 (9.3)	18 (8.5)		52 (9.0)	23 (12.0)	13 (12.9)		13 (6.3)	5 (4.5)	

### Specialty choice (Table 5)

Female physicians choose surgical fields less often and more often gynecology & obstetrics and pediatrics. Physicians with children more often choose primary care, have not yet decided on a specialty and are less inclined to choose anesthesiology.

### Workplace (Table 5)

Compared to male physicians, females work less often at university hospitals and research institutions, and more often in family medicine practice and in other medical fields. Compared to physicians with children, physicians without children tend to work more often at cantonal and university hospitals, and less often in other medical fields.

### Career aspirations (Table 5)

Female physicians more often aspire to a career as a hospital doctor without a senior position and less often to a hospital or academic career, or have yet to reach a decision on their career. Physicians with children aspire more often to a career as a family physician and less often to a hospital career. Compared to females without children, females with children more often opt for a medical career in a private practice and less often for a hospital career.

*In summary*, the results show that female physicians tend to be less advanced in their professional career and choose less prestigious medical specialties and career paths. Parenthood exacerbates these disadvantages, especially in females. That means that parenthood has to an extent a gender-differentiated effect.

## Discussion

Within the framework of our prospective SwissMedCareer Survey [13], the present paper reports on data acquired in the fifth questionnaire survey in 2009, seven years after cohort doctors' graduation from medical school. It focuses on gender-specific careers, especially on the impact of parenthood, and not on the various career stages between the beginning and the end of residency (T1 - T5).

### Employment

As reported in other studies [6,11,20-22], and as has also been found in the present study, female physicians with children work reduced hours, mainly to enable them to fit in domestic responsibilities. Although male physicians with children may also opt for part-time work [11], the male participants in our study mostly work full-time, regardless of whether they are parents or not. However, it is still difficult for males to find a part-time job [23]. Medicine in Switzerland seems to be a professional field in which traditional gender roles are not questioned to the same degree as in some other Western countries [6]. It has to be assumed that many mothers and fathers still think that childrearing is mainly the mother's responsibility.

### Career-related factors

As found in a previous study [14], female physicians, irrespective of whether they are parents or not, demonstrate lower objective career success scores in terms of publications, grants, scholarships, and research activities. This result can be traced back to the fact that women physicians aspire less often to an academic career than their male counterparts, findings that are also reported by other authors [3,24,25]. There are several reasons for the absence of women in academia: for instance, a lack of role models as well as rigid career paths, the latter being responsible for the incompatibility of work and family life [3,26]. Women often anticipate career obstacles in connection with parenthood and abstain from a prestigious career [5,20]. This also applies to their specialty choice: female physicians are significantly under-represented in prestigious surgical specialties [1,27-30]. From a constructive point of view, it has to be considered that the career success scale used only measures one aspect of career success in aiming for an academic career path. However, even for a leading position in a hospital, an academic degree (professorship) is often required in Switzerland.

When it comes to subjectively assessed career success, a more differentiated pattern emerges: Compared to the other groups, it is female physicians with children who assess their career success at the lowest level. This is a realistic assessment taking into account fewer hours of work and interruptions to training due to childbearing. Female physicians without parenting obligations assess their subjectively rated career success as high as their male colleagues; even so, they score lower in relation to the criterion of objective career success. As described, objective career success mainly means scientific success.

The satisfaction with one's own career does not vary between male and female physicians, irrespective whether they have children or not. However, physicians with children, regardless of their gender, tend to be less satisfied with their own career than those without children. This means that parenthood does not only have an impact on subjective career success, but also on an individual's satisfaction with his or her own career. It has to be assumed that the work-family conflict may be compounded to the extent that it has a negative impact on satisfaction with one's own career. Other authors also mentioned these influences [31,32].

Female doctors, especially those with children, more frequently aspire to work in a private general or specialist practice which allows for working flexible hours. This trend is also noticed in Great Britain [1,28,33]. It is furthermore striking that twice as many female as male study participants have not yet decided on their career path, although they are in their 8th year after graduation. It has to be assumed that female doctors anticipate difficulties in combining work and family obligations [20]. For this reason, their career planning strategy is often less well mapped out than is the case with male doctors [34], although career planning at an early stage represents a crucial factor, especially for female physicians wishing to start a family. This aspect should be addressed in mentoring programs.

### Mentoring

As shown in several studies [35-37], mentoring is a key factor in career support and success. In the present study female physicians, irrespective of whether they are parents or not, reported to have a mentor less often and to receive less career support in the sense of networking than male physicians. In order to set up mentorship, a mentee has to take the initiative and address an experienced senior physician higher up the hierarchy [38]; it is well-known that females are often reluctant to put themselves forward as proactive individuals [14,39]. Another reason is the absence of female role models who are able to act as mentors for female physicians. The "old boys' network" is known to play an important role in career support and females rarely profit from it [35].

A further point worth mentioning is that female and male respondents with children have less mentoring experience than those without children. It may be assumed that the demands of parenthood in terms of time will mean sacrifices in relation to the time needed for professional networking; this is often relegated to off-duty hours or to conferences.

Similar findings are evident in relation to factors 'satisfaction with own career' and 'satisfaction with career support'. Physicians with children are less satisfied than those without a family. Presumably physician parents suffer from work-family conflicts [32]. To summarize, having a family is, to some extent, at the expense of one's professional career.

### Work-Life balance

The cohort doctors were asked what kind of work-life pattern they aspire to in the future. As already seen in relation to careers, female physicians - and even more so if they have children - are less career-oriented and opt for part-time or a three-phase career path (work - family break - returning to the workplace). Not only females, but also parenting physicians of both genders are seeking a satisfying balance between their professional career and their personal life. These findings reflect the change in lifestyle aspired to by the younger generation of physicians: they want medical careers and working practices organized in such a way as to enable them to lead a "normal" life [1,10,33].

### Life-satisfaction

Overall, females are more satisfied than males, especially in the domains 'friends', 'leisure activities' and 'income'. As expected, physicians with children are more satisfied with family life, but this is the only difference of any significance in multivariate analysis. There is no interaction between gender and parenthood, i.e. no combined effect has been found to be engendered by these two factors. To point out an interesting detail, females appear to have lower expectations as regards enhanced satisfaction with 'income'. In fact, at this stage of their postgraduate training, there is generally-speaking no evidence of earnings being gender-biased.

In summary, significant differences in relation to gender are to be found in career-related factors and, to a smaller degree, in life satisfaction. However, the greatest gender-related difference in the work-life balance aspired to lies in the fact that females more often consider part-time work or a break in their professional career to discharge family obligations. This result reflects the fact that female physicians have a distinctly different scheme of life to their male colleagues.

### Gender segregation and gender equity

Although the profile of the medical profession has changed, the findings of the present study indicate that career paths in medicine are still gender-biased to the disadvantage of female physicians. Horizontal and vertical gender segregation can be discernible. Horizontal segregation is evident from the differences in choice of specialty. Males choose specialties such as surgery or cardiology which are more prestigious and provide higher income. Females are over-represented in specialties such as pediatrics, psychiatry or gynecology & obstetrics, specialties which carry lower prestige and income. The vertical gender segregation is reflected in the medical hierarchy. The higher positions in academia and hospital medicine are mainly held by male physicians.

There are a number of factors indicating that gender equity is far from being realized in medicine. Many young women choose to be trained in medicine because they want to treat patients. They often do not attach the same importance as men do to climbing the career ladder, achieving a high income or a powerful and influencing position. This attitude implies that females are often reluctant to put themselves forward and to appear proactive. The way they network is also different. Empathizing with a person is more important for females than whether a senior professional has major influence in the medical field. For this reason female physicians do not enjoy the same level of career support as males.

Anticipating the difficulties they are likely to encounter in combining a professional career with family obligations, females often do not plan their career in the same goal-oriented way as men. Such attitudes and behavior emerge as serious obstacles. To date, the idea of holding a senior position on a part-time basis has unfortunately not been readily accepted. Instead of fighting for career and employment conditions that fit into the female physicians' biography, they seem instead to be adapting to the existing male-dominated structures.

The present study has *strengths and limitations*. The issues of the impact of gender and parenthood on a professional career have not been studied in such detail and with such extensive sampling as has been the case in our study. Because the influence of these two factors is so extensive, we have limited the data analyses as regards gender and parenthood. A further strength is the high participation rate over a seven year period. Our report relates to a somewhat homogeneous sample in terms of age and professional status. Therefore, the findings cannot be extended to other academic professions and generalizations are not possible.

## Conclusion

The results of the present study reflect the deep-rooted gender role stereotypes in a well-educated younger generation of physicians. It is a matter of concern that medicine, at least in Switzerland, still seems to be a professional field that attracts persons with traditional attitudes. As more physicians seek a work-life and work-family balance, medicine will be challenged to develop creative models for integrating physicians with these lifestyle aspirations; failure will mean the loss of that segment of the workforce. In the coming years, Switzerland as well as Germany will be faced with a major lack of younger physicians to fill the gap produced by the retiring generation. To enhance the attractiveness of (clinical) medicine and retain physicians, especially females, in their chosen career path, structural conditions of medical institutions will need to be adapted using structured residency programs, providing mentorships, flexitime, less rigid career structures and child-care facilities. These factors will contribute to retaining physicians in their chosen career paths and to enhancing their life satisfaction [11,32,40].

## Competing interests

The authors declare that they have no competing interests.

## Authors' contributions

BBF is principal investigator. BBF, MS, RK and CB designed the study. MS and RK conducted the statistical analyses. All seven authors were involved in interpreting the data, BBF drafted the manuscript, which was critically revised by the other authors. All the authors read and approved the final manuscript.

## Pre-publication history

The pre-publication history for this paper can be accessed here:

http://www.biomedcentral.com/1472-6963/10/40/prepub
